# LUMPY: a probabilistic framework for structural variant discovery

**DOI:** 10.1186/gb-2014-15-6-r84

**Published:** 2014-06-26

**Authors:** Ryan M Layer, Colby Chiang, Aaron R Quinlan, Ira M Hall

**Affiliations:** 1 Department of Biochemistry and Molecular Genetics, Center for Public Health Genomics, University of Virginia, Charlottesville, VA 22908, USA; 2 Department of Public Health Sciences, University of Virginia, Charlottesville, VA 22908, USA; 3 Center for Public Health Genomics, University of Virginia, Charlottesville, VA 22908, USA

## Abstract

Comprehensive discovery of structural variation (SV) from whole genome sequencing data requires multiple detection signals including read-pair, split-read, read-depth and prior knowledge. Owing to technical challenges, extant SV discovery algorithms either use one signal in isolation, or at best use two sequentially. We present LUMPY, a novel SV discovery framework that naturally integrates multiple SV signals jointly across multiple samples. We show that LUMPY yields improved sensitivity, especially when SV signal is reduced owing to either low coverage data or low intra-sample variant allele frequency. We also report a set of 4,564 validated breakpoints from the NA12878 human genome. https://github.com/arq5x/lumpy-sv.

## Background

Differences in chromosome structure are a prominent source of human genetic variation. These differences are collectively known as structural variation (SV), a term that encompasses diverse genomic alterations including deletion, duplication, insertion, inversion, translocation or complex rearrangement of relatively large (for example, >100 bp) segments. While SVs are considerably less common than smaller-scale forms of genetic variation such as single nucleotide polymorphisms (SNPs), they have greater functional potential due to their larger size, and they are more likely to alter gene structure or dosage.

Our current understanding of the prevalence and impact of SV has been driven by recent advances in genome sequencing. However, the discovery and genotyping of SV from DNA sequence data have lagged far behind SNP discovery and genotyping because they are fundamentally more complex. SVs vary considerably in size, architecture and genomic context, and read alignment accuracy is compromised near SVs by the presence of novel junctions (that is, breakpoints) between the ‘sample’ and reference genomes. Moreover, SVs generate multiple alignment signals, including altered sequence coverage within duplications or deletions (read-depth), breakpoint-spanning paired-end reads that align discordantly relative to each other (read-pair), and breakpoint-containing single reads that align in split fashion to discontiguous loci in the reference genome (split-read) [1,2]. These diverse alignment signals are difficult to integrate and most algorithms use just one. Other methods use two signals, but to our knowledge these limit initial detection to one signal and use the second to add confidence, refine breakpoint intervals, or genotype additional samples [3-7].

An approach that integrates multiple signals allows for more sensitive SV discovery than methods that examine merely one signal, especially when considering heterogeneous samples and/or low coverage data, because each individual read generally produces only one signal type (for example, read-pair or split-read, but not both). The impact of improved sensitivity is particularly acute in low coverage datasets or in studies of heterogeneous cancer samples where any given variant may only be present in a subset of cells. However, even with high coverage data, integration of multiple signals can increase specificity by allowing for more stringent criteria for reporting a variant call.

## Results

Here, we present a novel and general probabilistic SV discovery framework that naturally integrates multiple SV detection signals, including those generated from read alignments or prior evidence, and that can readily adapt to any additional source of evidence that may become available with future technological advances.

### Overview of LUMPY

**Figure 1:**
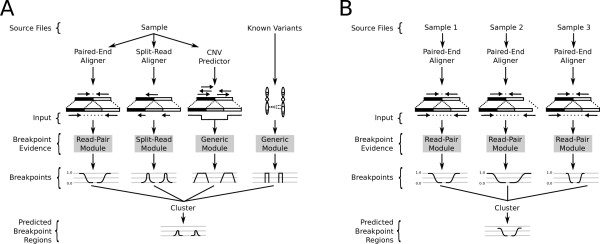
**The LUMPY framework for integrating multiple structural variation signals. (A)** A scenario in which LUMPY integrates three different sequence alignment signals (read-pair, split-read and read-depth) from a genome single sample. Additionally, sites of known variants are provided to LUMPY as prior knowledge in order to improve sensitivity. **(B)** A single signal type (in this case, read-pair) that is integrated from three different genome samples. We present these as example scenarios and emphasize that multi-signal and multi-sample workflows are not mutually exclusive. CNV, copy number variation.

Our framework is based upon a general probabilistic representation of an SV breakpoint that allows any number of alignment signals to be integrated into a single discovery process (Materials and methods). We define a breakpoint as a pair of bases that are adjacent in an experimentally sequenced ‘sample’ genome but not in the reference genome. To account for the varying level of genomic resolution inherent to different types of alignment evidence, we represent a breakpoint with a pair of probability distributions spanning the predicted breakpoint regions (Figure 1; Materials and methods). The probability distributions reflect the relative uncertainty that a given position in the reference genome represents one end of the breakpoint.

Our framework provides distinct modules that map signals from each alignment evidence type to the common probability interval pair. For example, paired-end sequence alignments are projected to a pair of intervals upstream or downstream (depending on orientation) of the mapped reads (Figure 1A). The size of the intervals and the probability at each position is based on the empirical size distribution of the sample’s DNA fragment library. The distinct advantage of this approach is that any type of evidence can be considered, as long there exists a direct mapping from the SV signal to a breakpoint probability distribution. Here, we provide three modules for converting SV signals to probability distributions: read-pair, split-read, and generic. However, we emphasize that our framework is readily extensible to new signals that may potentially result from new DNA sequencing technologies or alternative SV detection approaches. The read-pair module maps the output of a paired-end sequence alignment algorithm such as NOVOALIGN (C Hercus, unpublished) [8] or BWA [9], the split-read module maps the output of a split-read sequence alignment algorithm (for example, YAHA [10], BWA-SW [11], or BWA-MEM [5]), and the generic module allows users to include signals that do not have a specific module implemented such as prior knowledge of known SV (for example, 1000 Genomes [12]), and output from copy number variation (CNV) discovery tools (for example, CNVnator [13]).

Once the evidence from the different alignment signals is mapped to breakpoint intervals, overlapping intervals are clustered and the probabilities are integrated (see Materials and methods for details). Any clustered breakpoint region that contains sufficient evidence (based on user-defined arguments) is returned as a predicted SV. The resolution of the predicted breakpoint regions can be improved by trimming the positions with probabilities in the lower percentile of the distribution (for example, the lowest 0.1%).

It is well established that variant calling is improved by integrating data from multiple samples [5,6,14,15]. The LUMPY framework naturally handles multiple samples by tracking the sample origin of each probability distribution during clustering (Figure 1B; Materials and methods). As an example of a typical analysis, LUMPY can identify SVs in a whole-genome, 50X coverage paired-end Illumina dataset from the NA12878 CEPH individual in 12.2 hours using 8 Gb of memory using a single processor (Materials and methods). Given that these performance characteristics are comparable to existing approaches for SNP and indel detection, and that there is an approximately linear relationship between data volume, time and memory usage, we anticipate that simultaneous analysis of tens and eventually hundreds of human genomes will be possible with LUMPY using commodity hardware.

We implemented LUMPY in an open source C++ software package (available at [16]) that is capable of detecting SV from multiple alignment signals in BAM [17] files from one or more samples.

### Performance comparisons

We compared LUMPY’s performance to three other popular and actively maintained SV discovery packages: GASVPro [4], DELLY [3] and Pindel [18]. These algorithms were selected due to their widespread use and inclusion in large-scale projects such as The Cancer Genome Atlas (GASVPro) and 1000 Genomes (DELLY and Pindel). Moreover, Pindel was one of the first published SV discovery tools, and GASVPro and DELLY both consider a secondary SV signal along with paired-end alignments (read-depth and split-read, respectively). Both GASVPro and DELLY have also demonstrated substantial improvement over other popular SV tools such as Breakdancer [19] and HYDRA [20].

**Figure 2:**
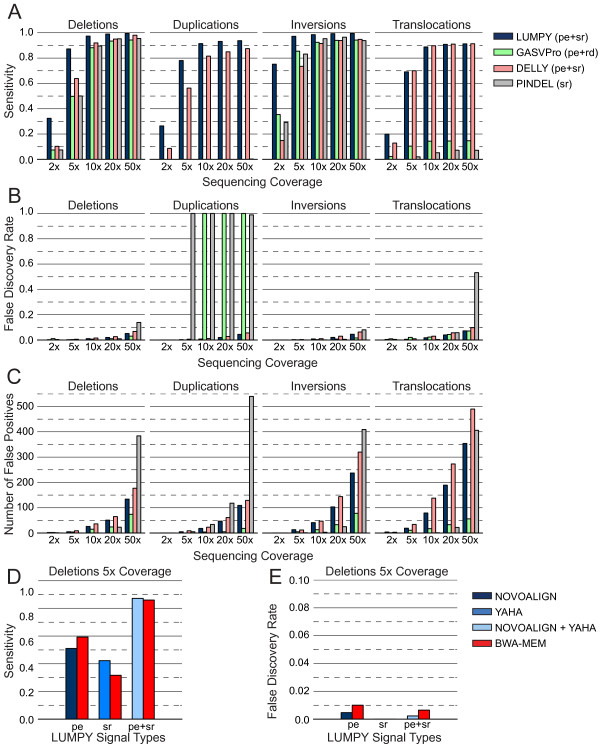
**Performance comparison using homozygous variants of various structural variation types.** We simulated a genome with SVs by embedding 2,500 deletions, tandem duplications, inversions or translocations in random locations in the human reference genome. We then simulated sequence data from the altered genome with varying levels of sequence coverage. The performance measurements for LUMPY and DELLY were based on paired-end (pe) and split-read (sr) alignments, GASVPro considered pe and read-depth (rd), and Pindel considered sr alignments. **(A)** Sensitivity for each tool. LUMPY was the most sensitive in most cases, and had a marked improvement at lower coverage. DELLY detected three more translocations than LUMPY at 20X, at the expense of 93 more false positives. **(B)** The corresponding FDR for each tool. LUMPY’s FDR was low in all but the highest coverage cases. GASVPro and Pindel did not support tandem duplications, but false calls were made in some cases, which resulted in a 100% FDR. **(C)** The absolute number of false positive calls. LUMPY had a high number of false positives in some cases, but these are counterbalanced by a higher number of true positives **(A)**. **(D,E)** To determine the impact that sequence alignment strategies had on SV detection accuracy, LUMPY’s sensitivity **(D)** and FDR **(E)** are shown when predicting deletions at 5X coverage via different alignment strategies from the simulations in (A-C). BWA-MEM produces both pe and sr alignment signals in a single alignment step, and serves as a basis of comparison to the NOVOALIGN (pe) and YAHA (sr) strategy. BWA-MEM provides better sensitivity than NOVOALIGN when using the pe signal alone, yet YAHA provides better sensitivity than BWA-MEM when using the sr signal alone. Sensitivity and FDR are roughly equivalent with either the BWA-MEM or NOVOALIGN/YAHA strategies when LUMPY integrates both alignment signals.

Detection performance was measured using both simulated data and previously published Illumina sequencing data from the widely studied NA12878 CEPH individual. The first simulation measured each tool’s basic detection capabilities in a prototypical scenario by simulating 2,500 homozygous variants from various SV classes at random genomic locations. The second simulation assessed the power of each tool to detect 5,516 known deletion variants present at varying allele frequencies within a mixed sample, as often occurs in heterogeneous tumors. Lastly, the analysis of SVs in the NA12878 genome assessed the performance of each tool on real data containing sequencing errors and other detection confounders that are difficult to simulate. In addition, the analysis of NA12878 (and her parents) measured the effect of considering multiple samples, prior SV knowledge, and third-party CNV predictions on LUMPY’s performance. In each case, we measured performance in terms of sensitivity and false discovery rate (FDR) by comparing the predicted SV breakpoints to either known breakpoints or split-read alignments from long-reads (Pacific Biosciences (PacBio) and Illumina Moleculo) that span the breakpoint. Predictions that either overlapped known variants or had sufficient long-read support were considered true positives, and all other predictions were considered false positives (Materials and methods).The simulated results were base on alignments generated by NOVOALIGN, and the NA12878 results were based on BWA alignments. In both cases, YAHA was used to generate split-read alignments. LUMPY was also tested using alternative read alignment pipelines using either BWA-backtrack or BWA-MEM for paired-end alignment, and BWA-MEM for split-read alignment (with roughly similar results; Figure 2D, E).

### Homozygous variants of different structural variation types

**Figure 3:**
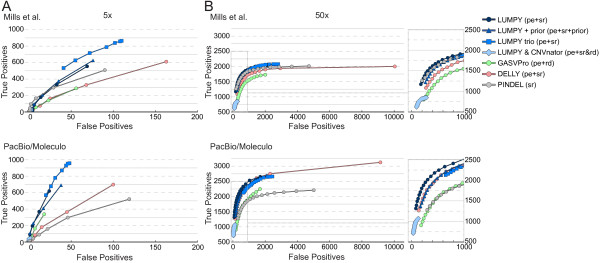
**Receiver operating characteristic** (**ROC) curves comparing deletion prediction performance in the NA12878 individual.** The relationship between true positive and false positive calls for deletions in the NA12878 genome is given for LUMPY, GASVPro, DELLY, and Pindel. Each point on a given tool’s ROC curve represents a minimum evidence support threshold ranging from 4 to 11 for 5X coverage and 4 to 20 for 50X coverage. Correctness was determined by two different methods: intersection with one of the 3,376 non-overlapping validated deletions from Mills *et al*. [12], or validation by PacBio/Moleculo data. **(A,B)** As in Figure 5, prediction performance was measured with both 5X mean genome coverage **(A)** and 50X coverage **(B)**. The curves are colored following the same convention described in Figure 5. LUMPY outperforms all other tools in all but one case. Pindel slightly outperforms LUMPY at higher-evidence thresholds in the 5X coverage case considering the Mills *et al*. truth set; we note that this is expected given Pindel was used by the 1000 Genomes Project as one of the tools to define this truth set. At the lower coverage, LUMPY’s performance is boosted by the inclusion of either prior evidence or NA12878’s parental genomes, but the read-depth signal is too weak to offer any improvement. The distinction between tools at 50X coverage is low, but it is expected given the coverage and quality of the data. At higher coverage, LUMPY is able to provide a high-confidence call set when considering read-depth, but priors and parental genomes have little added benefit. pe, paired-end; rd, read-depth; sr, split-read.

To assess the impact of coverage, SV type and SV size on algorithm performance, we first simulated a set of sample genomes that included 2,500 deletions, tandem duplications, inversions and translocations, randomly placed throughout the human genome (Additional file 1). Variants were created with SVsim, a tool that creates defined alterations to the reference genome (G Faust and I Hall, unpublished) [21]. For each SV type, the variant size ranged from 100 bp to 10 kb. We then used the WGSIM read simulator (H Li, unpublished) [22] to ‘sequence’ each simulated genome at 2X, 5X, 10X, 20X, and 50X haploid coverage.LUMPY was consistently more sensitive than the other algorithms across nearly all coverage levels and SV types (Figure 2A). DELLY had negligibly higher sensitivity (less than one percentage point) for translocations at higher coverage. LUMPY and DELLY were the only algorithms that detected all variant types; GASVPro and Pindel do not support detection of tandem duplications. (Note that tandem duplication was added to Pindel since we performed the analysis.) LUMPY’s superior sensitivity was most dramatic in lower coverage tests (<10X). For example, LUMPY detected 32.4% and 87.2% of all deletions at 2X and 5X coverage, respectively, whereas GASVPro detected 7.4% and 49.8%, DELLY detected 10.3% and 63.8%, and Pindel detected 7.4% and 50%. At best, LUMPY was 35.5 times more sensitive than Pindel for detecting translocations at 5X coverage (69.1% versus 2%). At worst, LUMPY was 0.009 times less sensitive than DELLY for detecting translocations at 2X coverage (69.1% versus 70.0%). At higher coverage (10 to 50X), LUMPY’s sensitivity advantage persisted; it ranged from 88.8% to 99.6% across all SV types, whereas GASVPro ranged from 14.3% to 94.3%; DELLY ranged from 81.2% to 98.0%; and Pindel ranged from 5.2% to 96.5% (excluding the SV types that GASVPro and Pindel are incapable of detecting).LUMPY’s FDR remained low (less than 4%) in all but the highest coverage cases (Figure 2B), and there was only one instance where LUMPY’s FDR was more than two percentage points higher than the best performing tool (GASVPro’s FDR for inversions at 50X was 1.6% while LUMPY’s was 4.9%). In general, the FDR for LUMPY, GASVPro, DELLY, and Pindel increased as coverage increased, ranging from 0% to 7.2% for LUMPY, 0% to 7.1% for GASVPro, 1% to 10% for DELLY, and 0% to 53.2% for Pindel. These patterns suggest that coverage-based parameter tuning could be used to minimize FDR for all the tools (as in Figure 3). We also note that FDR calculations depend on the number of true positives, which vary widely across SV varieties (Figure 2C). In certain cases (for example, translocations), LUMPY had a far higher absolute number of false positives, but these were counterbalanced by a much higher number of true positives as well. Alternatively, in cases where a specific SV type is not supported and no true positive calls were made (that is, GASVPro and Pindel for tandem duplications), the FDR reached 100%.

### Heterogeneous tumor simulation

**Figure 4:**
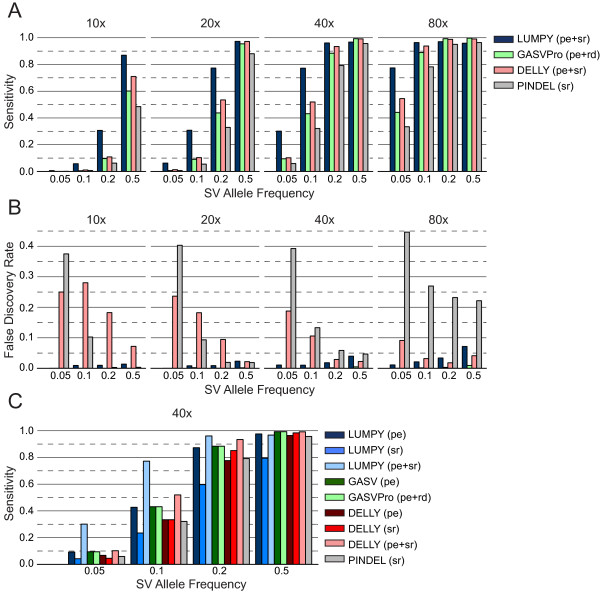
**Performance comparison for structural variations in a simulated heterogeneous tumor cell population.** To measure SV detection performance in the case of a heterogeneous tumor sample, we created a mock tumor genome by embedding 5,516 non-overlapping deletions identified by the 1000 Genomes Project into the human reference genome (build 37). Sequence reads were simulated separately from both the ‘tumor’ genome and the unaltered reference genome. We then mixed reads from both genomes in varying proportions to obtain simulated datasets representing a tumor cell population with different SV allele frequencies. Sequencing coverage levels are shown above each plot, and SV allele frequencies are shown beneath each plot. **(A)** Sensitivity for detecting SVs at varying allele frequencies and coverage levels. In all cases, LUMPY was more sensitive than GASVPro, DELLY, and Pindel, and showed a marked improvement when the coverage of the ‘tumor’ genome was low owing to either low sequence coverage or low SV allele frequency. In general, to achieve the same level of sensitivity as LUMPY, the other tools required twice the evidence from the ‘tumor’ genome. pe, paired-end; rd, read-depth; sr, split-read. **(B)** The FDRs for each tool at varying allele frequencies and coverage levels. The FDR for LUMPY was better than all other tools in all cases, with a notable improvement at lower SV allele frequencies. **(C)** The change in sensitivity when considering two SV detection signals versus a single signal alone is shown for the three tools at 40X coverage and at different SV allele frequencies. At low SV allele frequencies (for example, 5%), LUMPY’s use of two signals (that is, pe + sr) has a super-additive effect on sensitivity relative to either signal alone (that is, pe or sr), whereas the sensitivity of GASVPro and DELLY was either unchanged or modestly improved with one signal versus two.

Variant detection is especially challenging in tumor studies because biopsied samples generally include a mixture of abnormal and normal tissue, and because many tumor samples are composed of multiple clonal lineages defined by distinct somatic mutations. To assess the performance of our algorithm in this more realistic scenario where increased sensitivity is crucial, we simulated heterogeneous samples by pooling reads from an ‘abnormal’ genome and a ‘normal’ genome at varying ratios (Additional file 2). The source of the simulated abnormal genome was the human reference genome (build 37) modified (using SVsim) with 5,516 non-overlapping deletions identified by the 1000 Genomes Project [12], and an unmodified human reference genome was used to simulate the normal genome. As above, each genome was ‘sequenced’ using WGSIM, and the reads from the two genomes were combined in varying proportions to create a single heterogeneous sample. The ratio of the reads from the abnormal genome (SV allele frequency) varied between 5% and 50%, and the total coverage ranged from 10X and 80X. For example, to simulate a sample with a 5% SV allele frequency at 10X coverage, the abnormal genome was sequenced at 0.5X coverage and the normal genome at 9.5X coverage: when combined, the two sets of reads represent a single heterogeneous sample sequenced at 10X coverage.LUMPY was more sensitive than GASVPro, DELLY, and Pindel in nearly all cases, especially when the coverage of the abnormal genome was low owing to either lower coverage, low SV allele frequency, or both (Figure 4A). For example, at 10X coverage and 20% SV allele frequency LUMPY detects 30.7% of the SVs, whereas GASVPro, DELLY, and Pindel detect only 9.6%, 10.9%, and 6.2% of the SVs, respectively. This represents a 2.8-fold increase in sensitivity over the next best method. In general, to achieve the same level of sensitivity, GASVPro, DELLY and Pindel required roughly twice as much evidence as LUMPY (by either increased coverage or SV allele frequency). For example, at 20X coverage LUMPY detected 6.2% of variants present at 5% SV allele frequency, whereas GASVPro, DELLY, and Pindel required 10% SV allele frequency to achieve similar sensitivity (10.3%, 9.1%, and 5.5%, respectively). We note that this trend is also apparent across SV varieties in the previous homozygous test (Figure 2A). LUMPY had slightly lower sensitivity at higher coverage levels and SV allele frequencies, but was never more than four percentage points lower than the best performing tool. For example, at 80X coverage and 50% SV allele frequency LUMPY’s sensitivity was 95.9% and the best performing tool was GASVPro at 99.69% (a 3.79% difference).In all but the highest coverage and SV allele frequency case, the FDR for LUMPY was less than 4% (Figure 4B), and the FDR for GASVPro was less than 1% in all cases. For DELLY and Pindel, the FDR was particularly high when SV allele frequency was low. For example, at 10X coverage the FDR for DELLY was 3.4 times higher at 5% SV allele frequency than at 50% frequency, and the Pindel FDR was 111.9 times higher. At 20X coverage these differences were 10.9 and 21.5 times higher at 5% frequency than at 50% frequency for DELLY and Pindel, respectively. This is in contrast to LUMPY, where modest coverage-associated increases to FDR can likely be managed via parameter tuning, without significantly decreasing sensitivity.

### SV detection in the NA12878 genome

Although it is difficult to precisely measure the sensitivity and accuracy of SV predictions made from a real data set, it is also important to evaluate each tool’s performance when confronted with real data containing artifacts that are not easily captured by simulations (for example, PCR artifacts, chimeric molecules, reads from poorly assembled genomic regions, and so on). In this experiment we compared SV detection performance in the NA12878 individual by analyzing the Illumina Platinum Genomes dataset, which represents approximately 50X coverage of the NA12878 genome (European Nucleotide Archives; ERA172924). We additionally subsampled this dataset to approximately 5X coverage to assess SV detection in low coverage scenarios.

To estimate sensitivity and FDR, we compared predictions made by each tool to two truth sets: 1) 3,376 validated, non-overlapping deletions from the 1000 Genomes Project [12] (Additional file 3); and 2) 4,095 deletions that were detected by at least one tool in the 50X dataset, or that were reported by Mills *et al*. [12] (which used numerous SV detection tools), and that were validated by split-read mapping analysis of independent long-read sequencing data from PacBio or Illumina Moleculo platforms (Additional file 4). We expect that most *bona fide* SV calls will be validated by PacBio and/or Moleculo data given the read lengths (mean 3.7 kb and 1.8 kb, respectively) and coverage depth (mean 28.8X and 29.2X) of these datasets. The rationale for two truth sets is that although the 1000 Genomes callset is, to our knowledge, the most comprehensive set of deletions for NA12878, it still represents only a subset of the actual deletions in that individual’s genome. Analyses performed here have the benefit of higher quality sequencing data, longer reads and improved SV detection tools, and thus are likely to discover novel variants that were missed by Mills *et al*. [12]. Furthermore, since Pindel was one of the tools used to generate the 1000 Genomes callset [12], this truth set is biased against predictions made by LUMPY, GASVPro and DELLY. Monte Carlo shuffling of each SV callset resulted in validation efficiencies of less than 3%, indicating a low rate of spurious validation for false positive calls (Table 1; Materials and methods). Together, these two complementary approaches establish a catalog of high-confidence structural variants in the NA12878 genome.

**Table 1:** Long-read validation rates for each tool relative to randomly permuted data

**Method**	**Total calls**	**Observed validations (fraction)**	**Expected validations (fraction)**
**50X coverage**			
LUMPY (pe + sr)	4,347	2,653 (0.61)	37.9 ± 1.2 (0.009)
LUMPY (pe + sr + prior)	4,809	2,706 (0.563)	41.1 ± 1.3 (0.009)
LUMPY trio (pe + sr)	5,108	2,660 (0.521)	31.5 ± 1.1 (0.006)
LUMPY (pe + sr&rd)	1,355	1,114 (0.822)	5.4 ± 0.5 (0.001)
GASVPro	3,929	2,249 (0.572)	61.1 ± 1.5 (0.016)
DELLY	12,272	3,127 (0.255)	219.2 ± 2.9 (0.018)
Pindel	7,219	2,208 (0.306)	0.7 ± 0.2 (~0)
**5X coverage**			
LUMPY (pe + sr)	643	619 (0.963)	4.9 ± 0.4 (0.008)
LUMPY (pe + sr + prior)	840	785 (0.935)	4.3 ± 0.4 (0.005)
LUMPY trio (pe + sr)	1,006	958 (0.952)	4.1 ± 0.4 (0.004)
LUMPY (pe + sr&rd)	73	66 (0.904)	0.01 ± 0.02 (~0)
GASVPro	356	338 (0.949)	10.2 ± 0.6 (0.029)
DELLY	798	698 (0.875)	4.5 ± 0.4 (0.006)
Pindel	640	521 (0.814)	0.04 ± 0.04 (~0)

**Figure 5:**
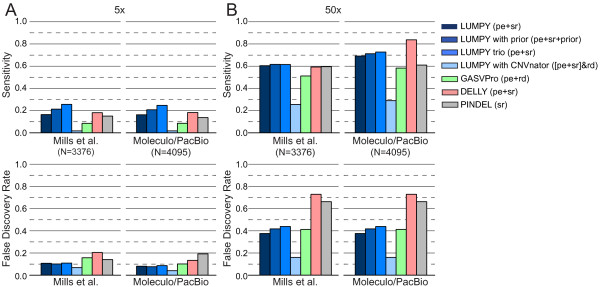
**Performance comparison of deletion detection in high and low coverage Illumina sequencing data from NA12878.** We analyzed an approximately 50X coverage dataset of the NA12878 genome from the Illumina Platinum Genomes dataset. We tested LUMPY’s performance under four different variant calling scenarios. First, ‘LUMPY (pe + sr)’ considered both paired-end (pe) and split-read (sr) alignments (using YAHA) from NA12878. Second, ‘LUMPY with prior’ considered pe and sr alignments as well as 1000 Genomes variants as prior evidence. Third, ‘LUMPY trio’ considered pe and sr alignments for NA12878 as well as alignments from her parents (NA12891 and NA12892). Lastly, ‘LUMPY with CNVnator’ integrated pe and sr alignments with copy number loss predictions made by CNVnator (read depth (rd)). DELLY considered pe and sr alignments, GASVPro considered pe alignments and rd, and Pindel considered sr alignments. Sensitivity and FDR were estimated using two truth sets: 3,376 non-overlapping validated deletions from Mills *et al*. [12], and 4,095 deletions that were predicted by at least one tool and validated by PacBio or Moleculo alignments. **(A)** SV detection sensitivity and FDR on a 5X coverage subsample of the original data. LUMPY pe + sr was more sensitive than both GASVPro and Pindel and had either an equivalent or better FDR. DELLY was more sensitive than LUMPY pe + sr, but also had a higher FDR. Prior evidence or parental genomes improved LUMPY sensitivity. Given the low coverage, the read-depth signal was weak and only a small number of CNVs clustered with paired-end or split-read calls. **(B)** SV detection sensitivity and FDR on the original 50X coverage data. LUMPY pe + sr, DELLY, and Pindel had similar sensitivity in the Mills *et al*. truth set, and in the PacBio/Moleculo truth set DELLY had the highest sensitivity and FDR. LUMPY pe + sr had the next best sensitivity and the lowest FDR.

**Figure 6:**
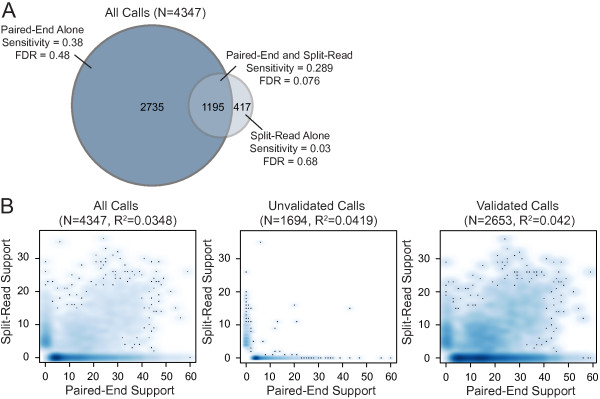
**Relationship between paired-end and split-read signals for the NA12878 callset. (A)** Venn diagram showing the total number of calls identified by paired-end alignments alone (left), by split-read alignments alone (right), or by both (center). Shown are the total number of calls, the sensitivity, and the FDR. Sensitivity and FDR are calculated precisely as in Figure 5. **(B)** Scatter plots showing the relationship between the number of split-reads (y-axis) and paired-end reads (x-axis) that identify each SV breakpoint in the entire callset (left), the unvalidated SV calls (center) and the validated SV calls (right). The number of variants in each category and the R^2^ values are shown above each plot. Note that one unvalidated call is not visible in these plots due to cropping; it was identified by 236 split reads and 0 paired-end reads.

Monte Carlo simulations were performed to assess the rate at which false positive SV calls are validated purely by chance using split-read mapping analysis of PacBio and Moleculo data. For each NA12878 deletion callset shown in Figures 5 and 6, deletion coordinates were shuffled 100 times (retaining the breakpoint interval sizes and total span of each deletion call), and validation experiments were conducted precisely as for real data. For each callset, we show the total number of deletion calls, the number of validated calls with the fraction validation in parentheses, and the number of validations expected by chance and the 95% confidence interval (with the expected fraction in parentheses) based on Monte Carlo simulations. pe, paired-end; rd, read-depth; sr, split-read.

A unique strength of LUMPY relative to other tools is the ability to consider different types of evidence from multiple samples. To demonstrate this capability, we included results for four different LUMPY configurations: 1) the standard configuration of read-pair and split-read signals from NA12878; 2) read-pair and split-read signals from NA12878, as well as prior knowledge (using LUMPY’s generic evidence module) of deletions discovered by the 1000 Genomes Project using low coverage whole genome sequencing (phase 1, release version 3); 3) read-pair and split-read signals from both NA12878 and her parents (NA12891 and NA12892); 4) read-pair and split-read signals from NA12878, plus CNV predictions based on read-depth analysis using CNVnator [13] (also using LUMPY’s generic evidence module). For clarity, this last dataset was filtered to only include LUMPY calls with both read-pair/split-read and CNV evidence. The last three calling strategies are unique to LUMPY and are intended to demonstrate both the ability and the benefit of including data from different samples, prior results, and other SV detection methods.

At 5X coverage (Figure 5A), LUMPY was more sensitive than both GASVPro and Pindel (16.4% versus 8.6% and 15% for the first (that is, 1000 Genomes) truth set and 16.3% versus 8.6% and 13.7% for the second (that is, the expanded ‘long-read’) truth set and had a better FDR (10.7% versus 15.7% and 14.1% for the first truth set and 8.1% versus 10.1% and 19.2% for the second truth set). While DELLY was more sensitive than LUMPY (18.1% for the first truth set and 18.3% for the second), it was at the expense of at least 60% higher FDR (20.4% and 13.4%). When LUMPY is provided with priors from the 1000 Genomes low coverage deletion calls, sensitivity increased to 21.3% and 20.8% with almost no change to FDR. Sensitivity is further improved to 25.6% and 24.7% when LUMPY performs simultaneous variant calling on NA12878 and her parents, with a similarly small effect on FDR, which clearly demonstrates the benefit of pooled variant calling on genetically related samples. Inclusion of read depth-based CNV calls as an additional input to LUMPY, followed by filtering to require CNV evidence, resulted in the lowest FDR among all tools (6.8% and 4.1%) but also greatly reduced sensitivity (1.7% in both cases); however, we note that CNVnator makes very few calls using low coverage data (n = 507), and thus this approach is better suited to high-coverage data (see below). As expected, the observed FDR for LUMPY, GASVPro, and DELLY was reduced (decreasing by 2.6, 5.6, and 7 percentage points, respectively) when lower coverage SV predictions were compared to the expanded ‘long-read’ truth set. In contrast, Pindel’s FDR increased by 5.2 percentage points; however, we note that this effect is expected considered that Pindel was used in part to create the 1000 Genomes Project truth set [12]. These results demonstrate that the expanded ‘long-read‘ truth set is a more comprehensive and unbiased reference; it will, therefore, be the basis for the remaining performance comparisons.At 50X coverage (Figure 5B) and considering solely the second (‘long-read’) truth set, the effect on performance was dramatic for all the tools except Pindel (as expected given the aforementioned bias). DELLY had the highest sensitivity at 83.8%, but this came at the expense of an extremely high FDR (72.9%). GASVPRO and Pindel exhibit intermediate levels of sensitivity (58.3% and 61%, respectively) and FDR (41.2% and 66.2%). LUMPY had the second highest sensitivity (69.1%) and the lowest FDR (37.5%) of any tool. When read depth-based CNV calls from CNVnator (n = 6,248) are included as input to LUMPY, the resulting FDR (15.3%) is nearly 2.5X lower than the next-best performing tool, although this comes at the cost of reduced sensitivity (29.5%). The main utility of such an analysis is therefore to greatly reduce lower FDR at calls exhibiting independent read-depth evidence, and to properly classify SVs that alter copy number. Similarly, if we consider a high confidence subset of 1,195 deletion calls that are detected by both paired-end and split-read signals, the FDR falls dramatically from 37.5% to 7.6%, but sensitivity also decreases from 69.1% to 28.9% (Figure 6A). This indicates that most variant calls exhibiting both paired-end and split-read signals are true positives, but that many variants are not well captured by one or the other signal, presumably due to local sequence features that inhibit accurate alignment. This interpretation is consistent with the observation that the strength of paired-end and split-read signals are not well correlated with each other (Figure 6B), which may account (at least in part) for LUMPY’s improved sensitivity over methods that consider the two signals sequentially. Taken together, the above results demonstrate that LUMPY provides significantly improved performance over other tools when one considers the trade-off between sensitivity and FDR.

In addition, the 4,095 deletions from our ‘long-read’ truth set (Additional file 4) combined with the 469 validated non-deletion variants reported by LUMPY (Additional file 5) provide what is, to our knowledge, the largest collection of validated SV breakpoints (N = 4,564) yet reported for a single ‘normal’ human genome. This dataset will be a valuable resource for future benchmarking studies.

### Improved performance in common usage scenarios

**Figure 7:**
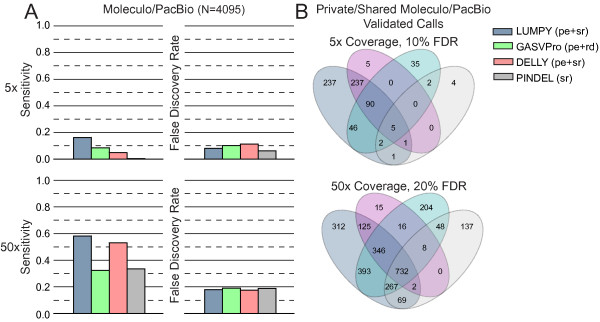
**Detection performance in the NA12878 individual when restricting false discovery rates.** We compared the performance of each tool in terms of sensitivity and novel variant discovery ability when considering only the subset of calls that meet a maximum FDR threshold. Using the results given in Figure 6, each tool’s FDR was calculated for each of the minimum-evidence settings used to generate the respective receiver operating characteristic (ROC) curves. This provided a mapping from the maximum FDR to the subset of calls that meet the associated minimum-evidence threshold for each tool. Sensitivity and FDR were estimated using the 4,095 deletions that were predicted by at least one tool and validated by PacBio or Moleculo alignments. **(A)** Sensitivity given a maximum FDR threshold. At 5X coverage, an FDR threshold of approximately 10% is achieved with a minimum of four alignments for LUMPY (8.1% FDR), four for GASVPro (10.1% FDR), six for DELLY (11.3% FDR), and nine for Pindel (6.3% FDR). An approximately 20% FDR at 50X coverage requires 8 alignments for LUMPY (18% FDR), 16 for GASVPro (19% FDR), 12 for DELLY (17.6% FDR), and 20 for Pindel (18.8% FDR). LUMPY had the highest sensitivity at both coverage levels and the relative improvement was most substantial at lower coverage. **(B)** Venn diagrams reflecting the absolute number of variants discovered uniquely and jointly among the different tools at both 10% FDR for 5X and 20% FDR for 50X. In both cases LUMPY found the most number of unique variants. The difference was most dramatic in the 5X coverage experiment, where only 46 out of 665 (6.9%) of the variants found among all four tools were missed by LUMPY. pe, paired-end; rd, read-depth; sr, split-read.

Importantly, although the comparisons presented in Figure 5 are based upon the choice of a single detection threshold chosen for each tool (Materials and methods), LUMPY outperforms other tools across a broad spectrum of thresholds (Figure 3), indicating that the framework itself - not arbitrary parameter choices - underlies LUMPY’s superior performance. It is also clear from this result that a more relevant measure of tool performance is the relative SV detection sensitivity achieved by each tool under parameter settings that result in an acceptably low FDR. We therefore varied the minimum-evidence parameter of each tool to select settings that achieved an FDR of approximately 10% at 5X coverage and approximately 20% at 50X coverage (Figure 7A). Using these settings, LUMPY was twice as sensitive as the next-best performing tool GASVPro (16.3% versus 8.6%) on low coverage 5X data, with DELLY at 4.9% and Pindel at 0.4% (Figure 7A). At 50X coverage, LUMPY was 1.1X more sensitive than the next-best performing tool, DELLY (58.2% versus 53%), with GASVPRO at 32.5% and Pindel at 33.5%. Therefore, when we control for FDR, LUMPY is significantly more sensitive than other tools on both low and high coverage data.

The above result has important practical implications: in the vast majority of genome sequencing-based study designs it is necessary to select tool parameter settings that constrain the FDR to acceptable levels, and an algorithm’s sensitivity under these conditions determines the number of true variants that can be discovered and the biological insights that can be gleaned. For example, at 5X coverage LUMPY finds 281 more true deletion variants than the next-best tool, GASVPro (619 versus 338), and finds 237 ‘novel’ deletions not found by any other tool (Figure 7B). At 50X coverage, LUMPY finds 232 more deletions than the next-best tool, DELLY (2,246 versus 2,014) and 312 ‘novel’ deletions not found by any other tool. In contrast, all other tools combined find only 46 variants not detected by LUMPY at 5X coverage, and only 428 at 50X coverage. Thus, although LUMPY finds only 196 truly novel validated deletions in NA12878 when one considers the entire set of calls made by all tools regardless of FDR (90 of which were not reported previously [12]), LUMPY detects hundreds of true variants that are not detected by the other methods when rational parameters that constrain FDR are used. Considering that our algorithmic approach is especially advantageous when variant coverage is low, and that clinically relevant somatic variants are often present at low allele frequencies due to tumor heterogeneity, LUMPY’s unsurpassed sensitivity at acceptable FDR levels translates directly to more comprehensive variant callsets and, hence, new biological insights.

## Discussion

We have developed a general probabilistic framework for SV discovery, and have demonstrated that our framework outperforms existing discovery tools across all SV types and coverage levels, and in both real and simulated human genome datasets. LUMPY’s performance improvements are especially pronounced when evidence is scarce, either due to low coverage data or low variant allele frequency. LUMPY therefore represents an important technological advance, particularly in the context of cancer genomics where sensitivity is crucial for identifying low frequency variants within heterogeneous tumor samples.

**Figure 8:**
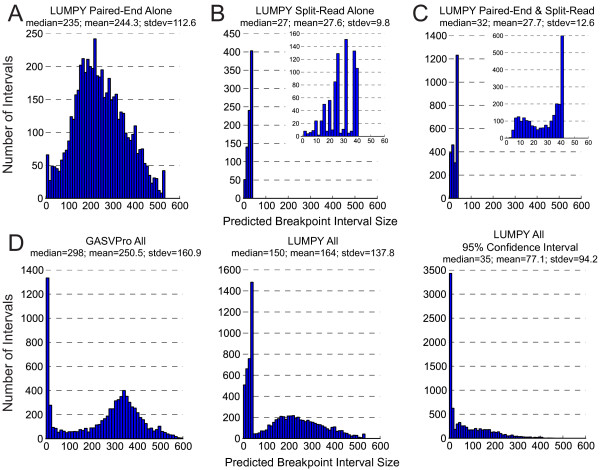
**Breakpoint interval size distributions for structural variation calls in NA12878.** LUMPY refines the location of a given breakpoint by taking the product of the probability distributions in the breakpoint’s evidence set. The shape of each distribution depends on the breakpoint uncertainty that is inherent to the evidence signal type (for example, the spatial uncertainty of breakpoints predicted by paired-end alignments is much higher than with split-read alignments). **(A)** The distribution of predicted breakpoint intervals for SV calls when using solely paired-end alignments. The variability in fragment size causes a significant amount of uncertainty in the paired-end signal, which results in a wide (over 500 bases for the NA12878 sample) distribution in the predicted breakpoint intervals. **(B)** The distribution of predicted breakpoint intervals for SV calls when using solely split-read alignments. Split-read alignments inherently have far less uncertainty in the predicted breakpoint location and, therefore, they yield a distribution with much lower variance. **(C)** The resulting breakpoint uncertainty distribution when both paired-end and split-read alignments are jointly considered. By taking the product of the distributions, the inherent breakpoint precision afforded by split-read alignments is not substantially diluted by paired-end alignments. **(D)** A comparison of the predicted breakpoint intervals reported by GASVPro (left) all LUMPY calls (center), and the 95% confidence interval for the LUMPY calls (right). Size distributions are not shown for DELLY or Pindel since they only report single base coordinates. stdev, standard deviation.

LUMPY’s high sensitivity is a direct consequence of combining multiple SV detection signals. LUMPY integrates disparate signals by converting them to a common format in which the two predicted breakpoint intervals in the reference genome are represented as paired probability distributions. SV prediction operations are then performed at this higher level. This novel approach has the key advantage that any SV detection signal can be integrated into the framework so long as a breakpoint probability can be assigned to each base pair in a candidate breakpoint region. Potential detection signals include paired-end and split-read alignments, alignments from assembled contigs, raw read-depth measurements, or CNVs detected by segmentation of read-depth, array comparative genomic hybridization or SNP array data. As we demonstrate using the NA12878 genome, inclusion of previously discovered SVs as priors can significantly enhance SV discovery sensitivity, while inclusion of third-party CNV calls as an input signal can significantly lower FDR, both of which are examples of the flexibility and generality of our framework. To facilitate integration, each evidence type can be assigned a different weight reflecting the user’s prior expectations. As sequencing technologies and SV detection strategies evolve, new sources of evidence can be easily incorporated without modifying the underlying logic of the SV detection algorithm itself; the sole requirement is the development of a new module that maps the SV detection signal to a paired probability distribution.In addition to facilitating signal integration, our use of probability distributions should, in theory, enable more accurate prediction of breakpoint positions. In most cases, not all coordinates within a predicted breakpoint interval are equally likely to be the breakpoint based on input data, and LUMPY’s use of probability distributions allows this spatial uncertainty to be propagated throughout the SV detection process. Although a detailed analysis of the spatial resolution provided by each SV detection tool is complicated by the variable ways in which different tools report breakpoints, our data suggest that LUMPY is matching if not exceeding the resolution of other tools. LUMPY reported significantly smaller breakpoint intervals than GASVPro (Figure 8), and although DELLY and Pindel report predictions at single base resolution, in order to achieve respectable validation rates it was necessary to ‘pad’ these coordinates to a similar interval size as those reported by LUMPY (Materials and methods). The use of probability distributions also allows LUMPY to report breakpoints at different levels of precision; by default, LUMPY reports the entire interval predicted to contain each breakpoint, the 95% confidence interval, and the most likely single base position. This feature will enable more accurate functional annotation of SV predictions. Following SV detection, LUMPY can report the final integrated probability distribution for each predicted variant to allow for comparison across studies. Alternatively, the final breakpoint probability distributions from one study could potentially be used as a source of prior evidence in another.

How could LUMPY’s performance be further improved? First and foremost, native support for read-depth data as an input signal should significantly improve performance at duplication and deletion variants. Here, we have incorporated read-depth information by converting the output of a copy number segmentation tool to breakpoint probability distributions, and providing these to LUMPY using the generic module (Figure 1A); however, we expect that more significant improvements will be possible using raw read-depth data. Second, for applications that require ultra-sensitive detection of known structural variants - such as low coverage population scale sequencing - LUMPY could be packaged with dataset priors reflecting the positions and allele frequencies of previously identified SVs. While we show that this is feasible using the existing LUMPY framework (Figure 5), we note that substantial improvements to sensitivity may require more comprehensive and accurate SV catalogs than are currently available. Third, LUMPY’s current evidence clustering logic is suboptimal for small insertion variants that are fully captured within a single read. These insertions are a special case because proper clustering of split-read signals requires that the size of the insertion event - as defined by read coordinates rather than the reference genome coordinates - be taken into account during clustering. Although this weakness is predicted to have minimal impact using short-read data, improving LUMPY’s behavior on insertion variants is a high priority given increasing use of long-read data. Finally, a major challenge for SV detection is distinguishing *bona fide* variants from false positives caused by alignment artifacts and other sources of error. In this respect, breakpoint probability distributions provide a highly quantitative source of information regarding the relative spacing of discordant and/or split alignments at a locus. By training on a set of known variants, it should be possible to derive a probabilistic measure of variant confidence that is based not only on the number of clustered reads, but also on the shape of the final integrated probability distribution. Alternatively, knowledge of the shape of ‘true’ breakpoint probability distributions could potentially be used as an objective function during read clustering.

In a more general sense, our approach for integrating SV detection signals - in essence, performing genome interval comparison operations using probability distributions rather than ‘flat’ features - could be useful for any application that involves comparison of genomic features whose exact coordinates are unknown, and whose positional uncertainty can be represented rationally in the form of a probability distribution. Rapid and efficient probabilistic comparisons could be enabled through extensions to existing interval-based software such as BEDTools [23].

## Conclusions

LUMPY’s superior sensitivity in these performance tests is a direct consequence of the fact that it simultaneously integrates multiple SV detection signals during SV discovery. The benefits of this approach are clear from the super-additive effect of combining read-pair and split-read signals within the LUMPY framework, relative to using either signal alone (Figure 4C). In contrast, although other tools such as GASVPro [4], DELLY [3], CNVer [7], CREST [6] and Genome STRiP [5] also exploit multiple SV detection signals, to our knowledge they first use one signal (that is, read-pair) to drive discovery and then refine and/or genotype candidates with a second signal (that is, split-read or read-depth). An intrinsic limitation of stepwise integration is that other available signals cannot increase the number of true positive SV calls beyond those candidates identified by the signal used for initial discovery. Consistent with this interpretation, inclusion of a second SV detection signal has little to no effect on DELLY’s or GASVPro’s sensitivity (Figure 4C) relative to using the primary read-pair signal alone.

## Materials and methods

We propose a breakpoint prediction framework that can accommodate multiple classes of evidence from multiple sources in the same analysis. Our framework makes use of an abstract breakpoint evidence type to define a set of functions that serve as an interface between specific evidence subtypes (for example, paired-end sequence alignments and split-read mappings) and the breakpoint type. Any class of evidence for which these functions can be defined may be included in our framework. To demonstrate the applicability of this abstraction, we defined three breakpoint evidence subtypes: read-pair, split-read, and a general breakpoint interface.

Since our framework combines evidence from multiple classes, it extends naturally to include evidence from multiple sources. The sources that can be considered in a single analysis may be any combination of evidence from different samples, different evidence subclasses from a single sample, or prior information about known variant positions. We refer to a given set of data as a breakpoint evidence instance, and assume that each instance contains only one evidence subtype and is from a single sample. To help organize the results of analysis with multiple samples or multiple instances for a single sample, each instance is assigned an identifier that can be shared across instances.

### Breakpoint

A breakpoint is a pair of genomic coordinates that are adjacent in a sample genome but not in a reference genome. Breakpoints can be detected, and their locations predicted by various evidence classes such as paired-end sequence alignments or split-read mappings. To support the inclusion of different evidence classes into a single analysis, we define a high-level breakpoint type as a collection of the evidence that corroborates the location and variety (for example, deletion, tandem duplication, and so on) of a particular breakpoint. Since many evidence classes provide a range of possible breakpoint locations, we represent the breakpoint’s location with a pair of breakpoint intervals where each interval has a start position, an end position, and a probability vector that represents the relative probability that a given position in the interval is one end of the breakpoint. More formally, a breakpoint is a tuple *b* = ⟨*E,l,r,v*⟩, where *b.E* is the set of evidence that corroborates the location and variety of a particular breakpoint; *b.l* and *b.r* are left and right breakpoint intervals each with values *b.l.s* and *b.l.e* that are the start and end genomic coordinates and *b.lp* is a probability vector where |*b.l.p*| = *b.l.e* – *b.l.s* and *b.l.p*[*i*] is the relative probability that position *b.l.s* + *i* is one end of the breakpoint (similar for *b.r*); and *b.v* is the breakpoint variety. Within the context of this method, breakpoint variety determinations are based on the orientation of the evidence. It is important to note that while a breakpoint may be labeled as a deletion when it contains evidence from a paired-end sequence alignment with a +/-orientation, the breakpoint may in fact be the result of some other event or series of complex events.

If there exist two breakpoints *b* and *c* in the set of all breakpoints *B* where *b* and *c* intersect (*b.r* intersects *c.r*, *b.l* intersects *c.l*, and *b.v* = *c.v*), then *b* and *c* are merged into interval *m*, *b* and *c* are removed from *B*, and *m* is placed into *B*. The evidence set *m.E* is the union of the evidence sets *b.E* and *c.E*.

A straightforward method to define breakpoint intervals *m.l* and *m.r* would be to let *m.l.s* = max(*b.l.s*, *c.l.s*) and *m.l.e* = min(*b.l.e*, *c.l.e*), similar for *m.r*. However, if a spurious alignment is merged into a set of genuine breakpoints, the resulting breakpoint interval can be ‘pulled’ away from the actual breakpoint. The impact of an outlier can be minimized or eliminated once the full set of corroborating alignments is collected for a given breakpoint, but collecting the full set is complicated by the fact that alignments are considered in order and outliers typically occur first. To account for this, we define a liberal merge process where *m.l.s* is the mean start position for the left intervals in *m.E*, and *m.l.e* is the mean end position for the left intervals in *m.E*, similar for *m.r*.

Once all the evidence has been considered, an SV call *s* (also a breakpoint) is made for each breakpoint *b* ∈ *B* that meets a user-defined minimum evidence threshold (for example, four pieces of evidence). The boundaries of the breakpoint intervals *s.l* and *s.r* are the trimmed product of the distributions of the left and right intervals in *b.E*. Let *s.l.s* = max({*e.l.s* | *e* ∈ *b.E*}), *s.l.e* = min({*e.l.e* | *e* ∈ *b.E*}), and *s.l.p*[*i*] = ∏_*e*∈*b.E*_*e.l.p*[*i*-*o*] where *o* is the offset value *e.l.s* - *s.l.s* (similar for *s.r*). The intervals *s.l* and *s.r* can then trimmed to include only those positions that are in the top percentile (for example, top 99.9% of values) based on a user-provided value. Given the liberal merge process, it is possible for *b.E* to contain non-overlapping distributions that would result in a zero-length product. In this case, we identify the maximum point among the sum of the distributions in *b.E*, any distribution not intersecting this point is removed, and the resulting subset processed normally. Regardless of the trimming value, LUMPY reports both the intervals that contain 95% of the resulting probability distribution and the maximum position of *s.l.p* and *s.r.p*. Summation is another option for calculating the combined distribution boundaries and values. In that case *s.l* and *s.r* are the trimmed mixture distributions of the left and right intervals in *b.E*. Let *s.l.s* = min({*e.l.s* | *e* ∈ *b.E*}), *s.l.e* = max({*e.l.e* | *e* ∈ *b.E*}), and *s.l.p*[*i*] = ∑_*e*∈*b.E*_*e.l.p*[*i*-*o*]. The value at *s.l.p*[*i*] (or *s.r.p*[*i*]) represents the level of agreement among the evidence in *b.E* that position *i* is one end of the breakpoint. While summation will give less precise breakpoint predictions, it can be a useful option when considering low-quality data.

### Breakpoint evidence

To combine distinct SV alignment signals such as read-pair and split-read alignments with the general breakpoint type defined above, we define an abstract breakpoint evidence type. This abstract type defines an interface that allows for the inclusion of any data that can provide the following functions: is_bp determines if a particular instance of the data contains evidence of a breakpoint; get_v determines the breakpoint variety (for example, deletion, tandem duplication, inversion, and so on); and get_bpi maps the data to a pair of breakpoint intervals.

To demonstrate the applicability of this abstraction, we defined three breakpoint evidence instances: paired-end sequencing alignments, split-read alignments, and a general breakpoint interface. Read-pair and split-read are the most frequently used evidence types for breakpoint detection, and the general interface provides a mechanism to include any other sources of information such as known variant positions or output from other analysis pipelines (for example, read-depth calls). As technologies evolve and our understanding of structural variation improves, other instances can be easily added.

### Paired-end alignments

Paired-end sequencing involves fragmenting genomic DNA into roughly uniformly sized fragments, and sequencing both ends of each fragment to produce paired-end reads ⟨*x*, *y*⟩, which we will refer to as ‘read-pairs’. Each read is aligned to a reference genome *R*(*x*) = ⟨*c*, *o*, *s*, *e*⟩, where *R*(*x*).*c* is the chromosome that *x* aligned to in the reference genome, *R*(*x*).*o* = +| - indicates the alignment orientation, and *R*(*x*).*s* and *R*(*x*).*e* delineate the start and end positions of the matching sequence within the chromosome. We assume that both *x* and *y* align uniquely to the reference and that *R*(*x*).*s* < *R*(*x*).*e* < *R*(*y*).*s* < *R*(*y*).*e*. While in practice it is not possible to know the position of read *x* in the sample genome (in the absence of whole-genome assembly), it is useful to refer to *S*(*x*) = ⟨*o*, *s*, *e*⟩ as the alignment of *x* with respect to the originating sample’s genome.

Assuming that genome sequencing was performed with the Illumina platform, read-pairs are expected to align to the reference genome with a *R*(*x*).*o* = +, *R*(*y*).*o* = - orientation, and at distance *R*(*y*).*e* - *R*(*x*).*s* roughly equivalent to the fragment size from the sample preparation step. Any read-pair that aligns with an unexpected configuration can be evidence of a breakpoint. These unexpected configurations include matching orientation *R*(*x*).*o* = *R*(*y*).*o*, alignments with switched orientation *R*(*x*).*o* = -, *R*(*y*).*o* = +, and an apparent fragment length (*R*(*y*).*e* - *R*(*x*).*s*) that is either shorter or longer than expected. We estimated the expected fragment length to be the sample mean fragment length *l*, and the fragment length standard deviation to be the sample standard deviation *s* from the set of properly mapped read-pairs (as defined by the SAM specification) in the sample data set. Considering the variability in the sequencing process, we extend the expected fragment length to include sizes *l* + *v*_*l*_*s*, where *v*_*l*_ is a tuning parameter that reflects spread in the data.

When *x* and *y* align to the same chromosome (*R*(*x*)*.c = R*(*y*)*.c*), the breakpoint variety can be inferred from the orientation of *R*(*x*) and *R*(*y*). If the orientations match, then the breakpoint is labeled as an inversion, and if *R*(*x*).*o* = - and *R*(*y*).*o* = + then the breakpoint is labeled as a tandem duplication. Any breakpoint with the orientation *R*(*x*).*o* = + and *R*(*y*).*o* = - is labeled as a deletion. When *x* and *y* align to different chromosomes (*R*(*x*)*.c ≠ R*(*y*)*.c*), the variety is labeled inter-chromosomal. At present, LUMPY does not explicitly support identification of insertions that are spanned by paired-end reads; however, if desired these can be identified in a post-processing step through assessment of ‘deletion’ calls.

To map ⟨*x*, *y*⟩ to breakpoint intervals *l* and *r*, the ranges of possible breakpoint locations must be determined and probabilities assigned to each position in those ranges. By convention, *x* maps to *l* and *y* to *r*, and for the sake of brevity we will focus on *x* and *l* since the same process applies to *y* and *r*. Assuming that a single breakpoint exists between *x* and *y*, then the orientation of *x* determines if *l* will be upstream or downstream of *x*. If the *R*(*x*).*s* = +, then the breakpoint interval begins after *R*(*x*).*e* (downstream), otherwise the interval ends before *R*(*x*).*s* (upstream).

The length of each breakpoint interval is proportional to the expected fragment length *L* and standard deviation *s*. Since we assume that only one breakpoint exists between *x* and *y*, and that it is unlikely that the distance between the ends of a pair in the sample genome (*S*(*y*).*e* - *S*(*x*).*s*) is greater than *L*, then it is also unlikely that one end of the breakpoint is at a position greater than *R*(*x*).*s* + *L*, assuming that *R*(*x*).*o* = +. If *R*(*x*).*o* = -, then it is unlikely that a breakpoint is at a position less than *R*(*x*).*e* - *L*. To account for variability in the fragmentation process, we extend the breakpoint to *R*(*x*).*e* + (*L* + *v*_*f*_*s*) when *R*(*x*).*o* = +, and *R*(*x*).*s* - (*L* + *v*_*f*_*s*) when *R*(*x*).*o* = -, where *v*_*f*_ is a tuning parameter that, like *v*_*l*_, reflects the spread in the data.

The probability that a particular position *i* in the breakpoint interval *l* is part of the actual breakpoint can be estimated by the probability that *x* and *y* span that position in the sample. For *x* and *y* to span *i*, the fragment that produced ⟨*x*, *y*⟩ must be longer than the distance from the start of *x* to *i*, otherwise *y* would occur before *i* and *x* and *y* would not span *i* (contradiction). The resulting probability is P(*S*(*y*).*e* - *S*(*x*).*s* > *i* - *R*(*x*).*s*) if *R*(*x*).*o* = +, and P(*S*(*y*).*e* - *S*(*x*).*s* > *R*(*x*).*e* - *i*) if *R*(*x*).*o* = -. While we cannot directly measure the sample fragment length (*S*(*y*).*e* - *S*(*x*).*s*), we can estimate its distribution by constructing a frequency-based cumulative distribution *D* of fragment lengths from the same sample that was used to find *L* and *s*, where *D*(*j*) gives the proportion of the sample with fragment length greater than *j*.

### Split-read alignments

A split-read alignment is a single DNA fragment *X* that does not contiguously align to the reference genome. Instead, *X* contains a set of two or more substrings *x*_*i*_…*x*_*j*_ (*X* = *x*_1_*x*_2_…*x*_*n*_), where each substring aligns to the reference *R*(*x*_*i*_) = ⟨*c*, *o*, *s*, *e*⟩, and adjacent substrings align to non-adjacent locations in the reference genome *R*(*x*_*i*_).*e* ≠ *R*(*x*_*i*_ + 1).*s* + 1 or *R*(*x*_*i*_).*c* ≠ *R*(*x*_*i*_ + 1).*c* for 1 ≤ *i* ≤ *n* – 1. A single split-read alignment maps to a set of adjacent split-read sequence pairs (⟨*x*_1_, *x*_2_⟩,⟨*x*_2_, *x*_3_⟩,…,⟨*x*_*n*_-1,*x*_*n*_⟩), and each pair ⟨*x*_*i*_,*x*_*i*_ + 1⟩ is considered individually.

By definition, a split-read mapping is evidence of a breakpoint and therefore the function is_bp trivially returns true.

Both orientation and mapping location must be considered to infer the breakpoint variety for ⟨*x*_*i*_, *x*_*i*_ + 1⟩. When the orientations match *R*(*x*_*i*_).o = *R*(*x*_*i*_ + 1).*o*, the event is marked as either a deletion or a tandem duplication. Assuming that *R*(*x*_*i*_).*o* = *R*(*x*_*i*_ + 1).*o* = +, *R*(*x*_*i*_).*s* < *R*(*x*_*i*_ + 1).*s* indicates a gap caused by a deletion and *R*(*x*_*i*_).*s* > *R*(*x*_*i*_ + 1).*s* indicates a tandem duplication. These observations are flipped when orientations *R*(*x*_*i*_).*o* = *R*(*x*_*i*_ + 1).*o* = -. When the orientations do not match *R*(*x*_*i*_).o ≠ *R*(*x*_*i*_ + 1).*o*, the event is marked as an inversion and the mapping locations do not need to be considered. When *x* and *y* align to different chromosomes, the variety is marked as inter-chromosomal. LUMPY does not currently attempt to identify insertions that are completely contained within a long read, but this will be supported in future versions. We note that this capability requires long-read aligners to report the number and order of alignments within a read (which is not formally supported in the current SAM format specifications).

The possibility of errors in the sequencing and alignment processes creates some ambiguity in the exact location of the breakpoint associated with a split-read alignment. To account for this, each alignment pair ⟨*x*_*i*_, *x*_*i*_ + 1⟩ maps to two breakpoint intervals *l* and *r* centered at the split. The probability vectors *l.p* and *r.p* are highest at the midpoint and decrease exponentially toward their edges. The size of this interval is a configurable parameter *v*_*s*_ and is based on the quality of the sample under consideration and the specificity of the alignment algorithm used to map the sequences to the reference genome.

Depending on the breakpoint variety, the intervals *l* and *r* are centered on either the start or the end of *R*(*x*_*i*_) and *R*(*x*_*i*_ + 1). When the breakpoint is a deletion *l* is centered at *R*(*x*_*i*_).*e* and *r* at *R*(*x*_*i*_ + 1).*s*, and when the breakpoint is a tandem duplication *l* is centered at *R*(*x*_*i*_).*s* and *r* at *R*(*x*_*i*_ + 1).*e*. If the breakpoint is an inversion, *l* and *r* are both centered either at the start positions or end positions of *R*(*x*_*i*_) and *R*(*x*_*i*_ + 1), respectively. Assuming that *R*(*x*_*i*_).*s* < *R*(*x*_*i*_ + 1).*s*, if *R*(*x*_*i*_).*o* = + then *l* and *r* are centered at *R*(*x*_*i*_).*e* and *R*(*x*_*i*_ + 1).*e*, otherwise they are centered at *R*(*x*_*i*_).*s* and *R*(*x*_*i*_ + 1).*s*. If *R*(*x*_*i*_).*s* > *R*(*x*_*i*_ + 1).*s*, then the conditions are swapped.

### Generic evidence

The generic evidence subclass provides a mechanism to directly encode breakpoint intervals using the BEDPE format [17]. BEDPE is an extension of the popular BED format that provides a means to specify a pair of genomic coordinates; in this case the pair represents the two breakpoint positions in the reference genome. This subclass extends our framework to include SV signal types that do not yet have a specific subclass implemented. For example, the set of variants that are known to exist in the population can be included in the analysis of an individual or variants that are known to exist in a particular type of cancer can be included in the analysis of a tumor. This signal can be included in the analysis by expanding the edges of the predicted intervals to create breakpoint intervals, and encoding these intervals in BEDPE format. Each BEDPE entry is assumed to be a real breakpoint (is_bp), the variety is encoded in the auxiliary fields in BEDPE (get_v), and the intervals are directly encoded in BEDPE (get_bpi).

### Performance comparisons

Both simulated and real datasets were used to compare the sensitivity and FDR of LUMPY to other SV detection algorithms (GASVPro, DELLY, and Pindel). Two types of simulations were performed: one in which homozygous variants of diverse varieties were introduced at random positions throughout the reference genome, and another in which a heterogeneous tumor sample was simulated by mixing reads from a modified ‘abnormal’ human reference genome (containing 1000 Genomes deletions) and an unmodified ‘normal’ human reference genome in varying proportions. We also used publicly available Illumina sequencing data of the NA12878, NA12891, and NA12892 individuals. Two scenarios were considered: the original 50X coverage files, and 5X subsamples of the original data sets.

In the case of the homozygous simulation, we used SVsim to create new versions of the human reference genome (build 37) containing 2,500 simulated variants of each variety. For deletions, tandem duplications and inversions we randomly placed 2,500 non-overlapping variants ranging from 100 bp to 10,000 bp in size. To simulate translocations, we randomly inserted 2,500 non-overlapping inter-chromosomal regions of 1,000 bp, derived from random donor sites in the reference genome. Although we note that the true variant variety in this case is actually an insertion, the inserted segment exceeds the insert size of the sequencing library as well as the read length, and thus the breakpoints formed by such insertions accurately simulate a translocation. Each simulated genome was sampled to 40X, 20X, 10X, 5X, and 2X coverage.

To simulate a heterogeneous tumor sample, we combined simulated reads from both a modified and unmodified version of the human reference genome (build 37). The modified genome was created using SVsim, and included 5,516 non-overlapping deletions identified by the 1000 Genomes Project. Each simulation combined reads from both the modified and unmodified genomes in varying proportions. We refer to the proportion of reads that were derived from the modified genome as the SV allele frequency. The simulated SV allele frequencies were 5%, 10%, 20% and 50%, and the simulated coverages were 10X, 20X, 40X, and 80X. For example, in the simulation with 5% SV allele frequency and 10X coverage, the modified genome was sampled at 0.5X coverage and the unmodified genome was sampled at 9.5X coverage. The two sets of reads are then pooled into a single 10X coverage sample.

For all simulations, WGSIM was used to sample paired-end reads with a 150 bp read length, a 500 bp mean outer distance with a 50 bp standard deviation, and default error rate settings. Paired-end reads were mapped to the reference genome with NOVOALIGN version V2.07.08, using the random repeat reporting and allowing only one alignment per read. From the NOVOALIGN output, all soft-clipped (≥20 bp clipped length) and unmapped reads were realigned with the split-read aligner YAHA using a word length of 11 and a minimum match of 15. The NOVOALIGN output was used as input to DELLY, GASVPro, and Pindel, and both NOVOALIGN and YAHA output were used as input to LUMPY. In all algorithms, the minimum evidence threshold was four. For LUMPY, the tuning parameters min_non_overlap was set to 150, discordant_z was set to 4, back_distance was set to 20, weight was set to 1, and min_mapping_threshold was set to 1. For GASVPro, LIBRARY_SEPARATED was set to all, CUTOFF_LMINLMAX was set to SD = 4, WRITE_CONCORDANT was set to true, and WRITE_LOWQ was set to true. For DELLY, map-qual was set to 1, and the inc-map flag was set. DELLY paired-end (pe) and split-read (sr) calls were combined into a single paired-end and split-read (pe + sr) callset by taking the union of the two sets where the split-read call was retained when a call was common to both sets. For Pindel, minimum_support_for_event was set to 4, all chromosomes were considered, and report_interchromosomal_events was set to true. Since the output of DELLY and Pindel are single-base-resolution intervals, we increased the size of those intervals to match the mean interval size of a similar LUMPY call. Any call that had split-read support (Pindel and DELLY sr calls) was expanded to a 28 bp interval, and any call that had only paired-end support (DELLY pe calls) was expanded to a 282 bp interval. The intervals for GASVPro were not modified since, like LUMPY, it reports an interval whose size is based on the supporting evidence.

For the real data, LUMPY, GASVPro, DELLY, and Pindel considered Illumina sequencing of the NA12878 individual. The original sequencing files were at 50X coverage and were used in the 50X experiments. The 5X experiments considered sequencing files that were created by subsamples 10% of the original paired-end alignments. For all the tools, only deletion predictions on chromosomes 1 though X were considered. All sequencing samples were retrieved from the European Nucleotide Archives (submission ERA172924), and were previously aligned using BWA. Soft-clipped (≥20 bp clipped length) and unmapped reads were realigned with the split-read aligner YAHA using a word length of 11 and a minimum match of 15. In addition to the NA12878 data, the LUMPY trio results also considered sequencing data from that individual’s parents, NA12891 and NA12892. The LUMPY prior result considered all 1000 Genomes variant calls using the generic evidence module. The LUMPY read-depth results considered all deletion calls made by CNVnator [13] for the NA12878 genome with a window size of 100 for the 50X coverage experiment and 1,000 for the 5X coverage experiment. The single-base-resolution regions predicted by CNVnator were extended upstream and downstream by one-half the window size before being considered by LUMPY. Each tool was run with the same options that were used in the simulation experiments, except the minimum mapping quality for LUMPY, GASVPro, and Pindel was increased to 10. Since Pindel uses paired-end reads differently than the other tools, the default mapping quality of 20 was used. Each call required support of at least four. In the LUMPY trio result, a call had to have support of four from at least one individual (NA12878, NA12891, or NA12892) and at least one piece of support from NA12878. The weight for the 1000 Genomes variant calls in the LUMPY prior result was set to 2. In the LUMPY read-depth result, a call had to have support from read-depth and paired-end or split-read.

For the identification of the first truth sets, the Mills *et al*. study [12] validated 14,012 deletions in NA12878 across 11 independent laboratories. Once duplicate predictions were removed, the first truth set contained 3,376 non-overlapping deletions. The SV breakpoints predicted by each algorithm were compared to the known variants. A true positive was defined as a variant call where the two breakpoint intervals reported by a given SV detection tool both intersect with the two breakpoints introduced in the reference genome by simulation, and where the SV types (for example, deletion) match. To account for varied spatial resolution among the tools, we pad the simulated breakpoint coordinates with 50 bp of bidirectional slop, such that each simulated variant is represented by two 100 bp breakpoint intervals.

The second truth set consisted of the 4,095 deletions called by at least one tool in the 50X dataset or by the 1000 Genomes Project [12], and that were also validated by long-read sequencing from PacBio or Illumina Moleculo data. Overlapping calls were merged by retaining the minimum shared interval. PacBio reads (median length, 880 bp; mean depth, 30X) were aligned to GRCh37 using BWA Smith-Waterman (bwa bwasw -b 5 -q 2 -r 1 -z 20 -w 500). Illumina Moleculo reads (median length, 3,012 bp; mean depth, 30X) were aligned to GRCh37 with BWA-MEM (bwa mem -t 1 -B 4 -O 6 -E 1 -M). These BAM files are available from the 1000 Genomes Project repository at [24,25], respectively.

Deletion calls were considered to be validated if supported by at least two non-duplicate PacBio split reads or at least one Moleculo split read. A supporting long-read is defined by the following criteria: 1) the long-read is split by the aligner such that at least 20 bp aligned to the flanking sequence on either side of the reported breakpoint; 2) the left and right intervals of the split long-read both intersect with the respective left and right intervals of reported breakpoint, allowing 5 bp of slop space on either side of the long-read breakpoint to account for microhomology or inserted sequence at the novel adjacency; 3) the strand orientation of the split long-read is consistent with the strand orientation at the reported breakpoint; 4) in the case of PacBio, where at least two non-duplicate long-reads are required to validate a call, the two long-reads must not only fill criteria 1 to 3, but must also overlap with each other within 5 bp of slop on either side.

We performed Monte Carlo shuffling of the callsets to estimate the number of spurious long-read validations due to random chance. We shuffled each callset 100 times using BEDTools shuffle while retaining identical interval sizes, variant spans, and number of variants for each iteration [23]. Regions of the genome that were excluded from the original analyses were also excluded from the shuffling (see ‘Excluded regions’ section below). We then performed long-read validation as described above to each shuffled callset, with less than 3% of shuffled calls validating (Table 1).

### Excluded regions

For structural variation detection with LUMPY and other tools, we excluded regions of the reference genome with consistently high sequencing depth over multiple individuals, since high depth is indicative of artifacts in the reference assembly. To define these regions, we first aligned the 17-member CEPH 1463 pedigree to the GRCh37 human reference genome using BWA-MEM 0.7.5a-r405 (bwa mem -t 32 -M -p) [26]. Each member of the pedigree was whole-genome sequenced from a PCR-free library to 50X coverage with 101 bp reads, and is publicly available through the Illumina Platinum Genomes project [27]. We used BEDTools v2.17.0 to generate a BED graph of aggregate per-base coverage from all 17 individuals [23]. The mode and standard deviation of the aggregate depth were calculated separately for the autosomes and sex chromosomes. Any regions with depth exceeding 2 * mode + 3 standard deviations were excluded from our analyses. (We chose to double the mode to allow inclusion of duplicated copy number variant regions.) Finally, the mitochondrial chromosome was excluded entirely. A BED graph of the excluded regions can be obtained at [28].

## Abbreviations

bp: base pair; CNV: copy number variation; FDR: false discovery rate; PacBio: Pacific Biosciences; PCR: polymerase chain reaction; SNP: single nucleotide polymorphism; SV: structural variation.

## Competing interests

The authors declare that they have no competing interests.

## Authors’ contributions

RL developed the algorithms, wrote the source code, performed the experiments and prepared the figures. CC performed the long-read SV validation experiments. AQ and IH conceived the framework, devised the experiments and co-advised the project. RL, AQ and IH contributed equally to the writing of the manuscript. All authors read and approved the final manuscript.

## Supplementary Material

Additional file 1 **This file contains the breakpoints used for the homozygous variant simulation.** The format is BEDPE. Each line has a ‘TYPE:’ field that indicates DELETION, DUPLICATION, INVERSION, or TRANSLOCATION. Click here for file

Additional file 2 **This file contains the breakpoints used for the heterogeneous tumor simulation.** The format is BEDPE. These deletions are based on the variants released by the 1000 Genomes Project in [29]. We selected non-overlapping deletions that were at least 50 bases long and successfully lifted over from build 36 of the human reference genome to build 37. Click here for file

Additional file 3 **This file contains the breakpoints used for the Mills *et al*. truth set. **The format is BEDPE, which is described by [30]. The breakpoints are the non-overlapping validated deletions observed in NA12878 and are based on the variants given in [31]. Click here for file

Additional file 4 **This file contains the breakpoint intervals for the deletion predictions that were made by LUMPY (pe + sr, trio, prior, pe + sr&rd), GASVPro, DELLY, Pindel, or the 1000 Genomes Project **[12]** and that were validated by long read alignments from PacBio and/or Illumina Moleculo sequencing.** The format is BEDPE, and the score field indicates the number of overlapping predictions. Note: in some cases one algorithm made two predictions that contributed to a single call. For example, there are two calls with a score of 8. In both cases Pindel contributed two very similar calls. Click here for file

Additional file 5 **This file contains the calls made by LUMPY for NA12878 with paired-end and split-read evidence that were also validated with PacBio/Moleculo data.** The format is BEDPE and the score field is the total amount of supporting evidence. This file contains extra fields that are described at [16]. Click here for file
